# Deletion of low-density lipoprotein-related receptor 5 inhibits liver Cancer cell proliferation via destabilizing Nucleoporin 37

**DOI:** 10.1186/s12964-019-0495-3

**Published:** 2019-12-27

**Authors:** Jinxiao Chen, Da Wo, En Ma, Hongwei Yan, Jun Peng, Weidong Zhu, Yong Fang, Dan-ni Ren

**Affiliations:** 1 Department of Plastic and Burn Surgery, Ninth People’s Hospital, Shanghai Jiaotong University, School of Medicine, 639 Zhi Zao Ju Road, Shanghai, 200011 People’s Republic of China; 2 Fujian Key Laboratory of Integrative Medicine on Geriatric, Academy of Integrative Medicine, Fujian University of Traditional Chinese Medicine, 1 Qiuyang Road, Minhou Shangjie, Fuzhou, 350122 Fujian China; 3 Clinical and Translational Research Center, Research Institute of Heart Failure Shanghai East Hospital, Key Laboratory of Arrhythmias of Ministry of Education, Tongji University School of Medicine, Shanghai, China

**Keywords:** LRP5, NUP37, Nuclear pore complex, Wnt/β-catenin signaling, Cancer cell proliferation

## Abstract

**Background:**

LRP5/6 are co-receptors in Wnt/β-catenin pathway. Recently, we discovered multiple β-catenin independent functions of LRP5/6 in tumor cells and in the diseased heart. Nucleoporin 37 (NUP37) is an important component of the nuclear pore complex (NPC), whose elevated expression is associated with worsened prognosis in liver cancer. Previous studies have shown that NUP37 interacted with YAP and activated YAP/TEAD signaling in liver cancer. Our preliminary findings showed a nuclear location of LRP5. We thus tested the hypothesis that LRP5 may act as a genuine regulator of YAP/TEAD signaling via modulating NUP37 in a β-catenin-independent way.

**Methods:**

We performed siRNA knockdown of LRP5, LRP6, or β-catenin in liver cancer HepG2 cells to determine the effect on tumor cell proliferation. Protein expressions and interaction between LRP5 and NUP37 were determined using immunoprecipitation and western blot analyses.

**Results:**

HepG2 cell proliferation was markedly inhibited by knockdown of LRP5 but not LRP6 or β-catenin, suggesting that LRP5 has a specific, β-catenin-independent role in inhibiting HepG2 cell proliferation. Knockdown of NUP37 by siRNA inhibited the proliferation of HepG2 cells, whereas overexpression of NUP37 reversed the decrease in cell proliferation induced by LRP5 knockdown. Immunoprecipitation assays confirmed that LRP5 bound to NUP37. Furthermore, LRP5 overexpression restored NUP37 knockdown-induced downregulation of YAP/TEAD pathway.

**Conclusions:**

LRP5 deletion attenuates cell proliferation via destabilization of NUP37, in a β-catenin-independent manner. LRP5 therefore acts as a genuine regulator of YAP/TEAD signaling via maintaining the integrity of the NPC, and implicates a therapeutic strategy in targeting LRP5 for inhibiting liver cancer cell proliferation.

## Background

Low-density lipoprotein-related receptors 5 and 6 (LRP5/6) are commonly regarded as Wnt coreceptors involved in activating Wnt/β-catenin pathway [1–3]. Upon binding to Wnt ligands, LRP5/6 cooperates with Frizzled to activate Wnt/β-catenin signaling pathway and subsequently prevent the ubiquitination and degradation of cytoplasmic β-catenin, thereby leading to the nuclear translocation of β-catenin and activation of Wnt target genes [4–6]. Recently, we reported that LRP5/6 could prevent Frizzled-regulated non-canonical pathway activation via directly binding to the Frizzled receptor [7], and established a novel working model on the roles of LRP5/6 in canonical and non-canonical pathways. Furthermore, we showed that Wnt inhibitors insulin-like growth factor binding protein 4 (IGFBP-4) and Dickkopf-1 (DKK1) played opposing roles in cardiac ischemia via differential targeting to LRP5/6 and β-catenin [8]. A separate study showed that LRP6 but not LRP5 deletion greatly promoted mTOR phosphorylation and acted as a major regulator of cardiomyocyte cell growth in a β-catenin-independent manner [9]. These studies demonstrated that LRP5/6 have various Wnt/β-catenin-independent physical and pathological functions that are important during adult homeostasis. However, the biological diversity and roles of LRP5/6 are yet to be fully elucidated.

The nuclear pore complex (NPC) is composed of roughly 34 different proteins termed nucleoporins (NUPs) that assemble together to form a large ~ 120 megadalton transport channel embedded in the nuclear envelope. Maintaining the integrity of the NPC is critical, which would otherwise impact the regulation and shuttling of numerous signaling proteins [10, 11]. Nucleoporin 37 (NUP37) is an indispensable component of the conserved NUP107–160 complex, which locates in the outer rings of the NPC and composes a major scaffold module of the NPC assembly [12, 13]. Recent studies have shown that upregulated expression of NUP37 in HCC acts as a positive regulator of YAP/TEAD signaling, thereby promoting cancer progression [14]. In addition, data from Human Protein Atlas showed that there was a negative correlation between the expression of NUP37 and patient survival rate [15]. Therefore NUP37 is regarded as a prognostic indicator for liver cancer, where high expression of NUP37 is associated with worsened patient prognosis.

HepG2 cells harbor a constitutively active mutant of β-catenin, which results in the over-activation of Wnt/β-catenin signaling [16]. Thus, we utilized HepG2 cells as an ideal model for studying the β-catenin-independent functions of LRP5/6. Here, we examined the effect of LRP5, LRP6, or β-catenin knockdown on liver cancer HepG2 cell proliferation. Previous studies have shown that NUP37 interacted with YAP and activated YAP/TEAD signaling by enhancing the interaction between NUP37 and YAP [14]. Accordingly, we explored the relationship between LRP5/6 and NUP37, and its effect on YAP/TEAD signaling. Our findings provide important insights into the roles of LRP5 and NUP37 in maintaining the integrity of the NPC and subsequent promotion of cancer progression in hepatocellular carcinoma (HCC). Taken together, we revealed the β-catenin-independent biological functions of LRP5 in inhibiting the proliferation of HepG2 cells.

## Materials and methods

### Cell proliferation and siRNA knockout assay

HepG2 cells were seeded on 35 mm dishes using Dulbecco’s modified Eagle’s medium (DMEM, Gibco, USA) and cultured at 37 °C in an incubator containing 5% CO^2^. Cells were transfected using RNAiMAX (Invitrogen, Carlsbad, CA, USA) with siRNAs for human LRP5 (HSS106156–1#, HSS106157–2#, HSS106158–3#), human LRP6 (HSS106153), human β-catenin (HSS102460), human NUP37 (HSS128152-a#, HSS128153-b#, HSS128154-c#) or negative control siRNA (Invitrogen) in OPTI-MEM (Invitrogen). Cells were counted and imaged 48 h after transfection for further analysis. Results are representative of at least three independent experiments.

### MTT and CCK8 assay

MTT and CCK8 cell proliferation and cell viability assays were performed to measure the viability and proliferation of HepG2 cells, according to the manufacturer’s instructions (MTT, C0009, Beyotime Biotechnology) and (CCK8, C0038, Beyotime Biotechnology). Briefly, cells were knock down by siRNAs after 48 h, then seeded in 96 well plates and cultured for another 72 h. MTT or CCK8 solution were then added to each well and incubated at 37 °C for 4 h for MTT, or 1 h for CCK8, prior absorbance measurements at 570 nm for MTT, or 450 nm for CCK8, using a microplate reader (SpectraMax® M5 Microplate Reader, Molecular Devices).

### Western blotting assay

Western blotting was performed as previously described [17]. Briefly, total protein was extracted using RIPA buffer (Beyotime Biotechnology), resolved on SDS-PAGE gels and transferred onto PVDF membranes. Following blocking with non fat dry milk in TBST, membranes were washed and incubated with primary antibodies for (anti-LRP5, #5731, CST; NUP37, ab220675, Abcam; anti-CTGF, #86641, CST; Flag-tag antibody and myc-tag antibody from Genscript) overnight at 4 °C. Membranes were then incubated with the appropriate HRP-conjugated secondary antibodies for 1 h at room temperature and detected with chemiluminescence using Immobilon Chemiluminescent HRP Substrate (Millipore). Results are representative of at least three independent experiments.

### Real-time PCR assay

Total RNA was extracted using TRIzol reagent (Takara Biotechnology, China) and reverse-transcribed to cDNA using a Prime Script II cDNA Synthesis Kit (Takara Biotechnology) according to the manufacturer’s instructions. Real-time quantitative PCR was performed with SYBR-Green master mix (Applied Biosystems, Foster City, CA, USA) in 96-well optical plates using a QuantStudio 6 Flex Real-Time PCR System (Thermo Fisher Scientific). GAPDH was used as the reference gene for determination of relative gene expressions. Results are representative of at least three independent experiments.

### Co-immunoprecipitation assay

Co-immunoprecipitation (co-IP) analyses were performed as previously described. Briefly, plasmids were transiently transfected into HEK293 cells for 48 h. Cells were lysed using NP-40 lysis buffer (Beyotime) and IP was performed using Anti-Flag M2 Affinity Gel (Sigma), or anti-c-Myc Agarose (Thermo Fisher Scientific) and mouse IgG Agarose as a negative control (Thermo Fisher Scientific). After incubation at 4 °C for 2 h, agarose was washed three times with TBST, then incubated with 100 μl 0.1 N NH_4_OH for 5 min. Following centrifugation, the resulting supernatant was collected and neutralized to pH 7.0 with using acetic acid. The samples were heated in 1 x reducing loading buffer at 95 °C for 5 min and subsequent Western Blot analysis was performed to examine protein binding. Results are representative of at least three independent experiments.

### Tumor implantation assay

All animal protocols were approved by the Animal Care and Use Committee of Fujian University of Traditional Chinese Medicine and followed the ARRIVE guidelines. LRP5/6 siRNAs, β-catenin siRNA, or control siRNA were transfected into HepG2 cells using RNAiMAX. After 48 h, a total of 1 × 10^6^ cells were subcutaneously injected into the lower flanks of severe combined immunodeficient/beige (SCID/bg) mice. Tumor diameters were measured with digital calipers and the tumour volume in mm^3^ was calculated using the formula: tumor volume = (width)^2^ × (length / 2).

### Reporter gene assay

Luciferase activity was examined using Luciferase Assay kit (Promega, E1980). Briefly, TOPFLASH reporter gene together with siLRP5/6 or Wnt3a construct was transfected in HEK-293 cells. c-fos/α-MHC-Luciferase reporter gene was transfected in HEK-293/AT1–293 cells following Angiotensin II stimulation after 24 h transfection using Fugene (Roche) in 48-well plates (3 × 10^4^ cells per well) for 48 h. Lysates of HEK293 cells were measured according to the manufacturer’s instructions. All data were normalized by Renilla Luciferase activity.

### Statistical analysis

All data were obtained from at least three independent experiments performed in duplicate. Data are presented as the mean ± SE of the mean (SEM). Graphs and statistical analysis were done using GraphPad Prism (version 6.01; GraphPad Software, Inc). All statistical analyses were analyzed using SPSS 16.0 software (SPSS, Inc., Chicago, IL, USA). *P*-values of less than 0.05 were considered statistically significant.

## Results

### Knockdown of LRP5 inhibits the proliferation of HepG2 cells

HepG2 cells harbor a constitutive active mutant of β-catenin with basal over-activation of Wnt/β-catenin pathway, which was verified using TOPflash reporter gene assay, which was upregulated 135-fold compared to HEK293 cells, a separate cell line without the active mutant of β-catenin (Additional file 1: Figure S1). We next examined the tumor formation and Wnt activity following siRNA knockdown of LRP5, LRP6, and β-catenin. SCID/bg nude mice injected with HepG2 cells following knockdown of β-catenin by siRNA had no effect on tumor formation, whereas knockdown of LRP5/6 led to significantly smaller tumor volume (Additional file 1: Figure S2), suggesting a β-catenin-independent role of LRP5/6 in regulating HepG2 cell proliferation. We further investigated the individual role of LRP5 and LRP6 using TOPflash reporter gene assay in HepG2 cells, which showed that Wnt activity was significantly affected by siRNA knockdown of LRP6, but not LRP5 in the absence or presence of Wnt3a (Additional file 1: Figure S3). Thus, we utilized HepG2 cells as an ideal model for studying the β-catenin-independent functions of LRP5.

**Fig. 1:**
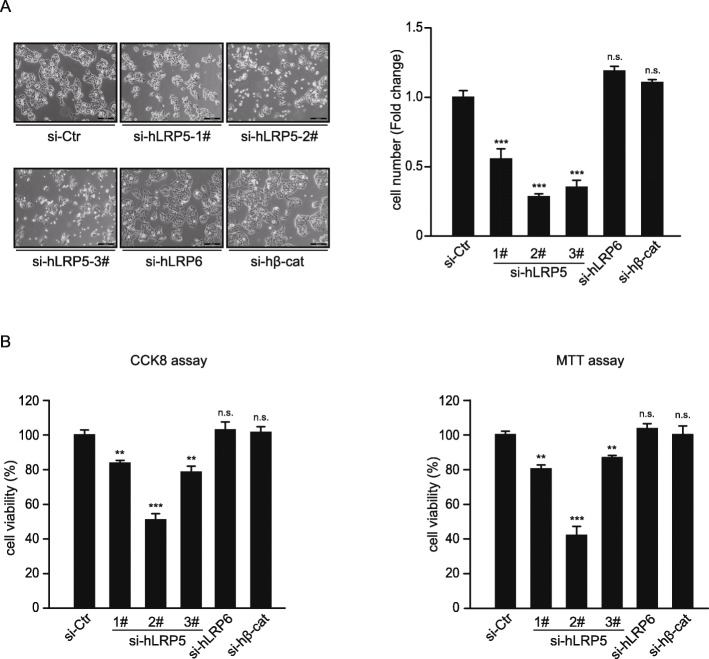
Knockdown of LRP5 inhibits the proliferation of HepG2 cells. **a** Representative image of HepG2 cells following siRNA knockdown of LRP5 (three distinct siRNA targeting different segment of LRP5 ORF), LRP6 or β-catenin for 48 h (left). *n* = 3. Quantification of total cell count. *** *P* < 0.001, n.s., no significance, compared to siRNA control cells (right). **b** CCK8 and MTT assays of cell proliferation and cell viability in HepG2 cells following siRNA knockdown of three distinct siRNA targeting LRP5 ORF. ** *P* < 0.01, *** P < 0.001, n.s., no significance, compared to siRNA control cells

LRP5 and LRP6 are plasma membrane receptors commonly thought to be involved in the activation of Wnt/β-catenin pathway. However, the role of LRP5 and LRP6 in liver cancer has not been fully investigated. We used RNA interference method with three distinct (siRNAs) in order to eliminate any ubiquitous off-target effects. Knockdown efficiency of all tested siRNAs, including LRP5, LRP6, β-catenin, and NUP37 were presented in Additional file 1: Figure S4 and S5. Knockdown of LRP5 with three distinct siRNAs (siLRP5–1#, siLRP5–2#, and siLRP5–3#) all significantly inhibited the proliferation of HepG2 cells, as well as a separate liver cancer cell line Huh7 cells (Fig. 1a and Additional file 1: Figure S6), indicating that knockdown of LRP5 may generally affect the proliferation of liver cancer cells. However, knockdown of LRP6 as well as β-catenin, the key effector protein in Wnt/β-catenin pathway had no obvious effects on HepG2 cell proliferation (Fig. 1a). Furthermore, MTT assay and CCK8 assay were performed to determine the effects of LRP5/6 knockdown on cancer cell viability and proliferation, which showed that knockdown with LRP5, but not LRP6 or β-catenin, significantly inhibited the cell viability and proliferation of HepG2 cells (Fig. 1b). These results indicated that LRP5 specifically inhibits HepG2 cell viability and proliferation, and furthermore, this effect is independent of Wnt/β-catenin pathway.

### Knockdown of LRP5 destabilizes NUP37

**Fig. 2:**
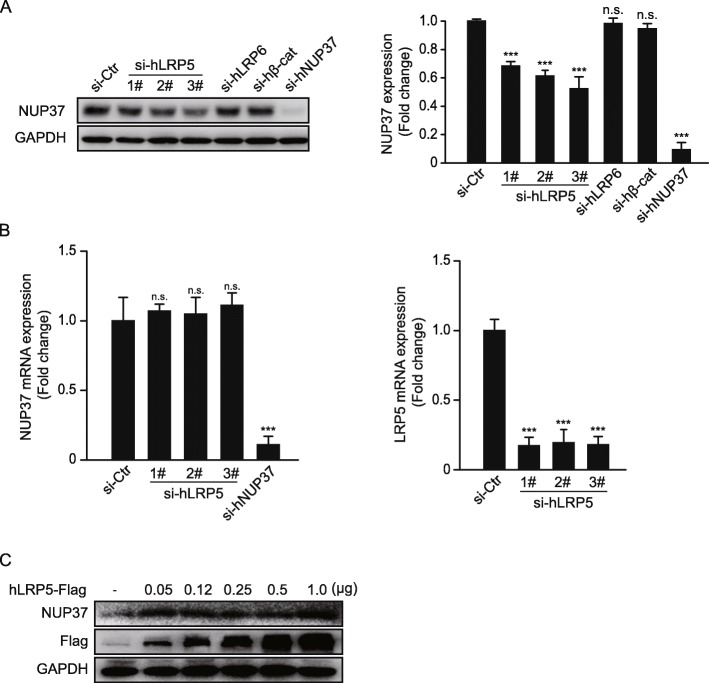
Knockdown of LRP5 destabilizes NUP37. **a** Western blot showing the expression of NUP37 following siRNA knockdown of LRP5 (three distinct siRNA targeting different segment of LRP5 ORF) or LRP6 or β-catenin for 48 h (left). si-hNUP37, positive control. *n* = 3. Densitometry analysis normalized with the loading control, GAPDH (right). **b** Real-time PCR analysis following siRNA knockdown of LRP5 (three distinct siRNA targeting different segment of LRP5 ORF), showing no significant changes in mRNA expression of NUP37 (left), si-hNUP37, positive control. n = 3. and significant down-regulation of LRP5 mRNA expression (right). n = 3. **c** Western blot analysis demonstrating that over-expression of LRP5 promotes stabilization of nuclear NUP37 in dose dependent manner, even at relatively low dosage. GAPDH, loading control

Knockdown of LRP5 markedly downregulated the expression of NUP37, but had no effect on its transcription level following treatment with three distinct siRNA targeted open reading frame (ORF) of LRP5, which indicated that the downregulation of NUP37 expression induced by knockdown of LRP5 occurs during the posttranslational modification stage (Fig. 2a and b). Furthermore, overexpression of LRP5 promoted the stabilization of nuclear NUP37, in a dose dependent manner (Fig. 2c).

### NUP37 interacts with LRP5 and promotes proliferation of HCC

**Fig. 3:**
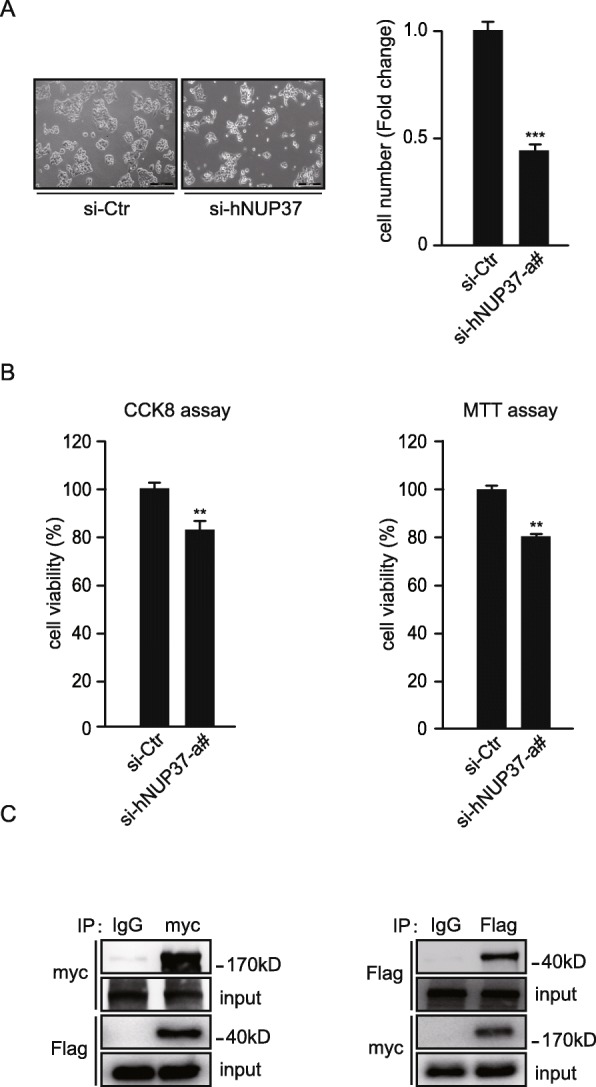
NUP37 interacts with LRP5 and promotes proliferation of HCC. **a** Representative image of HepG2 cells following siRNA knockdown of NUP37 for 48 h (left). n = 3. Quantification of total cell count, siRNA control cells were set as fold change of 1 (right). *** *P* < 0.001. **b** MTT assay and CCK8 assays of cell proliferation and cell viability in HepG2 cells following siRNA knockdown of NUP37. ** P < 0.01. **c** Co-IP assay demonstrating the binding between LRP5 with NUP37 in HEK293 cell line

Shuttling of signaling proteins between the cytoplasm and nucleus is tightly regulated by the NPC, which is composed of approximately 34 different NUPs that are packaged into a complex cylindrical structure. Previous studies have demonstrated that NUP37 is significantly upregulated in HCC clinical samples, and overexpression of NUP37 can promote proliferation of HCC cells via interacting with YAP and subsequently activating YAP/TEAD signaling [14]. Here, we further examined the effect on cell proliferation following knockdown of NUP37 in HepG2 cells. Indeed, similar to knockdown of LRP5, knockdown of NUP37 significantly inhibited the proliferation of HepG2 cells (Fig. 3a-c) and Huh7 cells (Additional file 1: Figure S6), as well as a non liver cancer cell line HEK-293 cells (Additional file 1: Figure S7). Interestingly, co-IP analysis demonstrated a strong binding affinity between LRP5 and NUP37 (Fig. 3c), suggesting the presence of a molecular regulatory mechanism between LRP5 and NUP37.

**Fig. 4:**
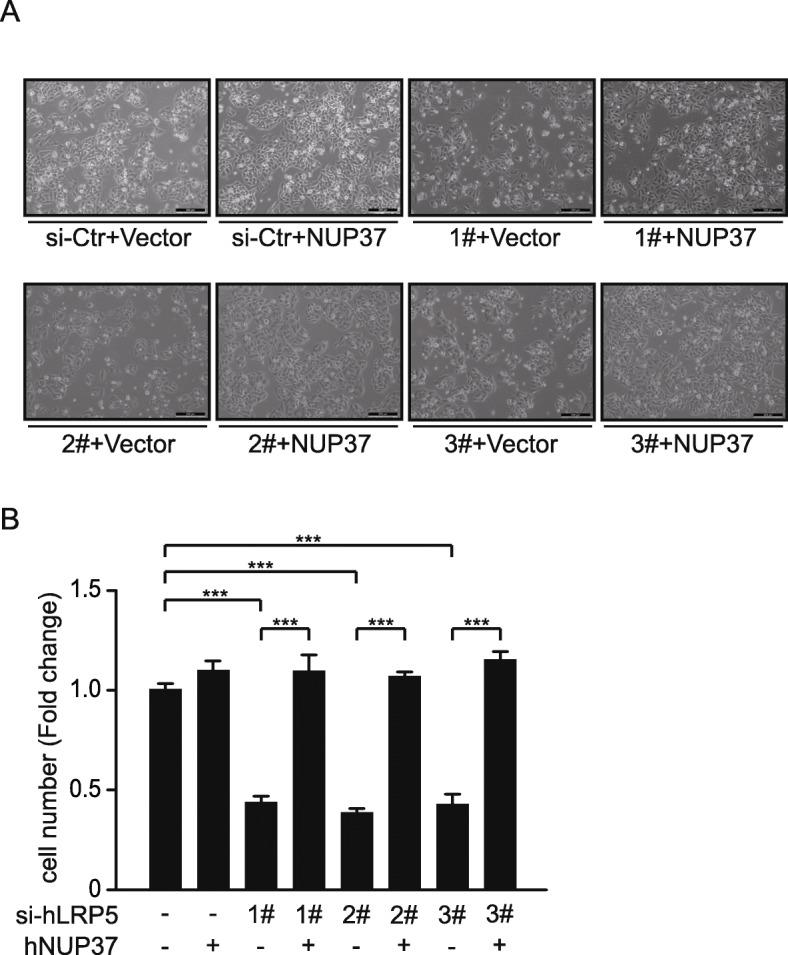
NUP37 overexpression reverses LRP5 knockdown-induced inhibition of cell proliferation. **a** Representative image of HepG2 cells following over-expression of NUP37, which completely restored all three LRP5 siRNA (#1, #2, #3) knockdown-induced inhibition of cell proliferation. **b** Quantification of total cell count, siRNA control cells were set as Fold change of 1. *** P < 0.001

In order to verify the fact that knockdown of LRP5 inhibited HepG2 cell proliferation via destabilization of NUP37, we examined whether overexpression of NUP37 could reverse this phenotype. Indeed, overexpression of NUP37 completely reversed all three LRP5 siRNA knockdown–induced inhibition in HepG2 cell proliferation, demonstrating that the LRP5 knockdown-induced inhibition on cell proliferation is specifically caused by destabilization of NUP37 (Fig. 4).

### LRP5 overexpression restores NUP37 knockdown-induced downregulation of YAP pathway

**Fig. 5:**
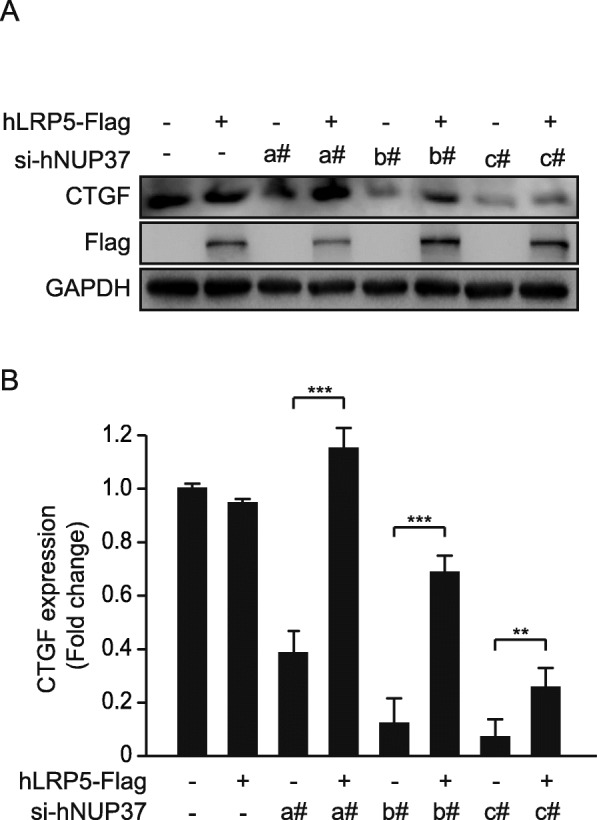
LRP5 overexpression restores NUP37 knockdown induced down regulation of YAP pathway. **a** Western blot showing knockdown of NUP37 with three distinct siRNA (#a, #b, #c) significantly decreases the expression level of YAP/TEAD signaling target gene CTGF, but restored by over-expression of LRP5, n = 3. **b** Densitometry analysis normalized with the loading control, GAPDH

As we demonstrated above, knockdown of LRP5 inhibited HepG2 cell proliferation via specific destabilization of NUP37. In order to clarify the underlying mechanisms, we used a YAP inhibitor CA3(CIL56), which significantly decreased HepG2 cell viability to ~ 65%, while knockdown of NUP37 using three different siRNAs all decreased HepG2 cell viability to ~ 85% compared to scrambled siRNA-treated cells (Additional file 1: Figure S8). Of note, we did not observe a further reduction in proliferation when YAP was inhibited in NUP37 knock-down cells (~ 65%), suggesting that knockdown of NUP37 specifically affects proliferation via YAP/TEAD signaling. Therefore, it is reasonable to suggest that overexpression of LRP5 may be able to rescue NUP37 knockdown-induced downregulation of YAP/TEAD signaling. Indeed, knockdown of NUP37 with three distinct siRNAs all significantly decreased the expression levels of YAP/TEAD signaling target gene CTGF, but were restored by the overexpression of LRP5 (Fig. 5). Taken together, our results demonstrated that LRP5 bound to and modulated the stability of NUP37, thereby maintaining the dynamic integrity of NPCs and subsequently promoting cancer progression in HCC. Thus, LRP5 acts as a genuine regulator of YAP/TEAD signaling, suggesting a promising therapeutic target for preventing HCC proliferation.

## Discussion

This is the first study which examined the physical and functional interaction between LRP5 and NUP37 in cancer. We demonstrated that knockdown of LRP5, but not LRP6 or β-catenin, markedly inhibited the proliferation of liver cancer HepG2 cells by destabilizing NUP37 and may result in the subsequent destruction of the NPC integrity. Moreover, overexpression of LRP5 restored NUP37 knockdown-induced downregulation of YAP/TEAD signaling target gene CTGF, suggesting that LRP5 acts as a genuine regulator of YAP/TEAD signaling, while also implicating LRP5 as a promising therapeutic target for preventing HCC proliferation.

Transport and translocation of signaling proteins between the cytoplasm and nucleus are tightly controlled by the NPC, a multi-protein channel located in a fusion pore between the outer and inner membranes of the nuclear envelope [11, 18–21]. The NPC is involved in the regulation of numerous cellular processes, such as gene expression and cell proliferation, but requires the synergistic cooperation between numerous components, and therefore, maintaining the integrity of the NPC is critical [10]. Our study revealed a previously unknown biological function of LRP5, whereby LRP5 binds to and stabilizes NUP37, one component of the outer rings of the NPC, which in turn maintains the integrity of the NPC. In contrast, LRP5 deficiency results in the destabilization of NUP37, and may lead to the destruction of the NPC integrity, which in turn causes dysregulation in the nuclear translocation of numerous signaling proteins, including those critically involved in the proliferation of liver cancer cells. Due to the fact that knockdown of NUP37 also significantly inhibited the proliferation of a separate liver cancer cell line Huh7, as well as non liver cancer cell line HEK-293, LRP5 may play a universal role in the modulation of NPC function via specific stabilization of NUP37. A recent study demonstrated that mutations in the genes encoding the outer ring components of the NPC, namely NUP107, NUP85, NUP133, and NUP160, were associated with the development of steroid-resistant nephrotic syndrome (SRNS), indicating that maintaining the integrity of the NPC is crucial [22]. Due to the critical role of LRP5 in maintaining the integrity of the NPC, thereby promoting cellular proliferation, LRP5 may be involved in promoting cancer progression in HCC.

LRP5 is mostly expressed on the cell membrane but also had visible nuclear expression (Additional file 1: Figure S9), whereas NUP37 is almost exclusively expressed in the cell nucleus. Thus, it is likely that LRP5 can enter the cell nucleus and interact with NUP37 within the nucleus. However, because the endogenous nuclear expression of LRP5 was low, our current study was unable to detect an endogenous interaction between LRP5 and NUP37. Nevertheless, due to the fact that LRP5 strictly controlled HepG2 cell fate via regulating NUP37, it is possible that under certain disease conditions, the entry of LRP5 into the nucleus may allow the detection of endogenous interaction between LRP5 and NUP37, which requires further investigation. Destabilization of NUP37 induced by the absence of LRP5 may occur via protein degradation, which is a coordinated process that involves protein recognition, attachment of multiple ubiquitin molecules and subsequent digestion by the 26S proteasome [23–27]. The recognition domain of the ubiquitin-proteasome complex in NUP37 may be covered when it binds to LRP5 in the normal state, but may also be uncovered upon dysfunction of LRP5 under diseased conditions, which requires further investigation.

## Conclusions

Our current study revealed a novel function of LRP5, which binds to and modulates the stability of NUP37 in a β-catenin-independent manner. Upregulation of NUP37 expression is critical for the progression of HCC via activation of YAP/TEAD signaling, where LRP5 may act as a genuine regulator of YAP/TEAD signaling via maintaining the integrity of the NPC. Our study implicates LRP5 as a promising therapeutic target for inhibiting liver cancer cell proliferation.

## Supplementary information

**Additional file 1 : Figure S1.** TOPflash assay showing the basal level of Wnt/β-catenin pathway activation between HCC cell line HepG2 cells and a non cancer cell line HEK293 cells. *n* = 3. **Figure S2.** Tumor formation assay following injection of HepG2 cells in SCID/bg mice after transient transfection with control, LRP5/6, or β-catenin siRNAs. *n* = 4. * *p* < 0.05, n.s. no significance, compared to si-Ctr. **Figure S3.** TOPflash assay showing the individual roles of LRP5 and LRP6 in regulating Wnt/β-catenin pathway. *n* = 3. **Figure S4.** Western blots showing the knockdown efficiency of all three LRP5 siRNAs, as well as LRP6 and β-catenin siRNAs. n = 3. GAPDH, loading control. **Figure S5.** Knockdown efficiency of all three NUP37 siRNAs as verified using real-time PCR assay (A), as well as western blot assay (B). n = 3. GAPDH, loading control. *** *p* < 0.001, compared to si-Ctr. **Figure S6.** MTT assay (A) and Photoimages (B) of Huh7 cell proliferation following knockdown of NUP37 or LRP5. n = 3. *** p < 0.001, compared to si-Ctr. **Figure S7.** Photoimages and quantification of HEK-293 cell proliferation following knockdown of NUP37. n = 3. ** *p* < 0.01, *** p < 0.001, compared to si-Ctr. **Figure S8.** MTT assay showing the decrease in cell proliferation following treatment with YAP inhibitor CA3(CIL56) in NUP37 knocked-down HepG2 cells. n = 3. ** p < 0.01, *** p < 0.001, compared to si-Ctr. **Figure 9.** Western blots showing the subcellular localization of LRP5 and NUP37. n = 3.

## Data Availability

The datasets used and/or analysed during the current study are available from the corresponding author on reasonable request.

## Abbreviations

CTGF: Connective tissue growth factor
DKK1: Dickkopf-1
HCC: Hepatocellular carcinoma
IGFBP-4: Insulin-like growth factor binding protein-4
LRP5: Low-density lipoprotein-related receptor 5
LRP6: Low-density lipoprotein-related receptor 6
NPC: Nuclear pore complex
NUP37: Nucleoporin 37
siRNA: small interfering RNA
TEAD: TEA Domain transcription factor
YAP1: Yes-associated protein 1
