# Supplementary material for: Deletion of low-density lipoprotein-related receptor 5 inhibits liver Cancer cell proliferation via destabilizing Nucleoporin 37
Source: Cell Commun Signal. 2019 Dec 27;17:174. doi: 10.1186/s12964-019-0495-3 (PMC6935199; doi:10.1186/s12964-019-0495-3)

Figure 1

A

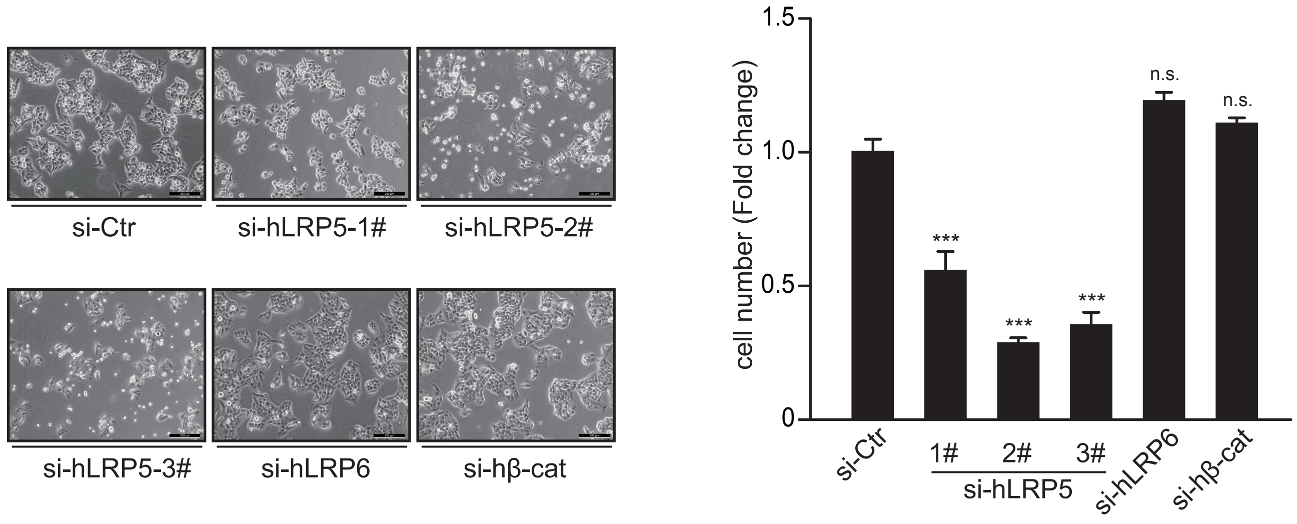
si-Ctr
si-hLRP5-1#
si-hLRP5-2#
si-hLRP5-3#
si-hLRP6
si-hβ-cat
cell number (Fold change)
1.5
1.0
0.5
0
n.s.
n.s.
***
***
***
si-Ctr
1#
2#
3#
si-hLRP5
si-hLRP6
si-hβ-cat

B

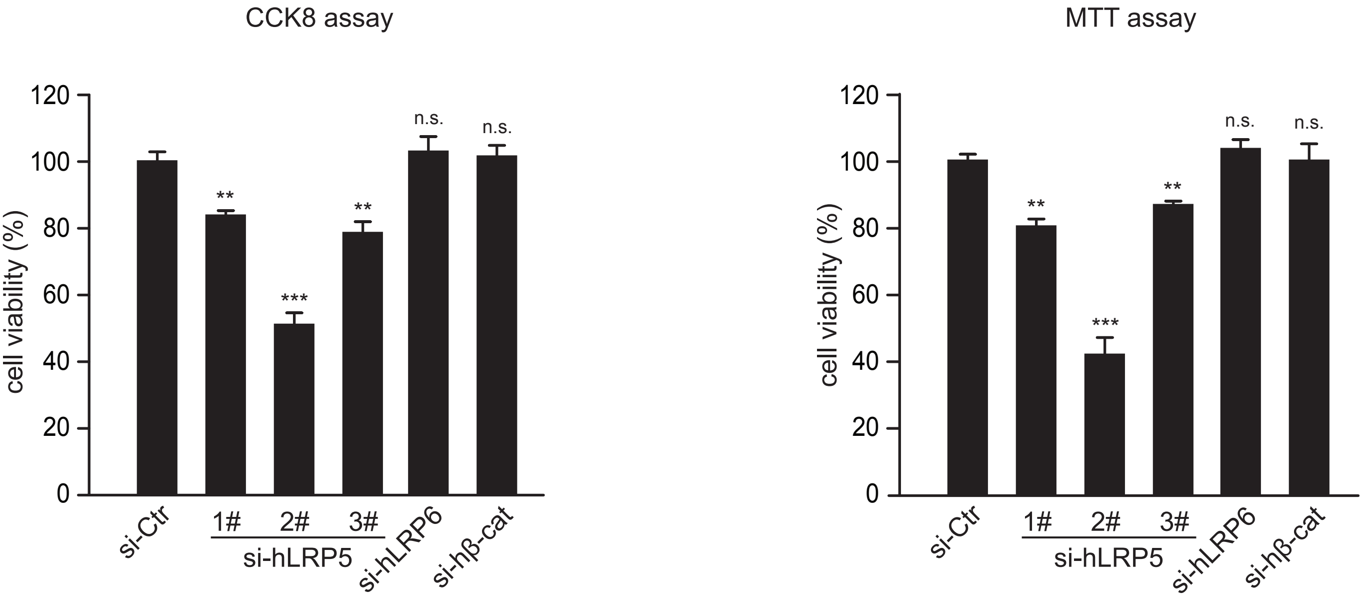
CCK8 assay
cell viability (%)
120
100
80
60
40
20
0
n.s.
n.s.
**
***
**
si-Ctr
1#
2#
3#
si-hLRP5
si-hLRP6
si-hβ-cat
MTT assay
cell viability (%)
120
100
80
60
40
20
0
n.s.
n.s.
**
***
**
si-Ctr
1#
2#
3#
si-hLRP5
si-hLRP6
si-hβ-cat

Figure 2

A

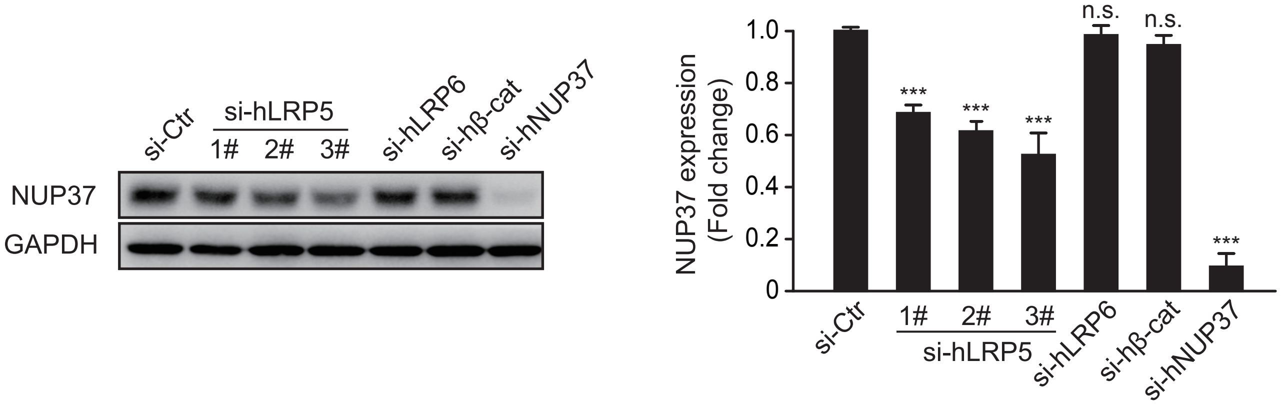
si-Ctr
si-hLRP5
1#
2#
3#
si-hLRP6
si-hβ-cat
si-hNUP37
NUP37
GAPDH
1.0
0.8
0.6
0.4
0.2
0
NUP37 expression
(Fold change)
***
***
***
n.s.
n.s.
***
si-Ctr
1#
2#
3#
si-hLRP5
si-hLRP6
si-hβ-cat
si-hNUP37

B

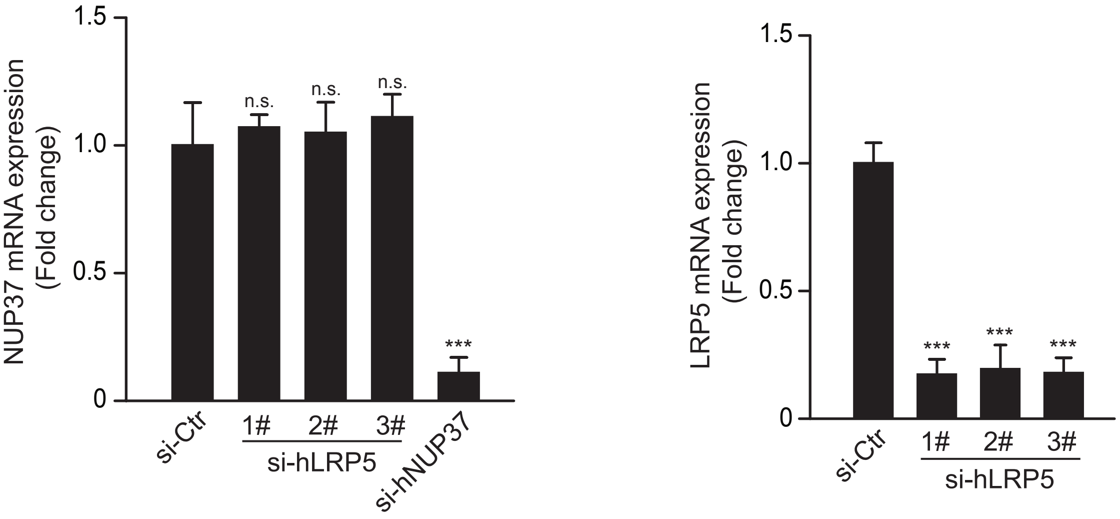
1.5
1.0
0.5
0
NUP37 mRNA expression
(Fold change)
n.s.
n.s.
n.s.
***
si-Ctr
1#
2#
3#
si-hLRP5
si-hNUP37
1.5
1.0
0.5
0
LRP5 mRNA expression
(Fold change)
***
***
***
si-Ctr
1#
2#
3#
si-hLRP5

C

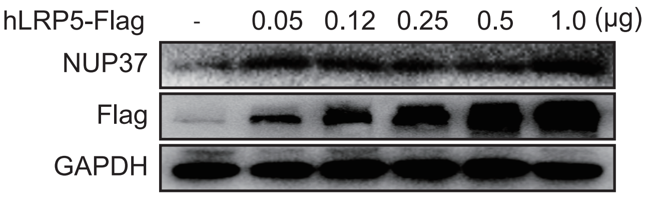
hLRP5-Flag
-
0.05
0.12
0.25
0.5
1.0 (μg)
NUP37
Flag
GAPDH

Figure 3

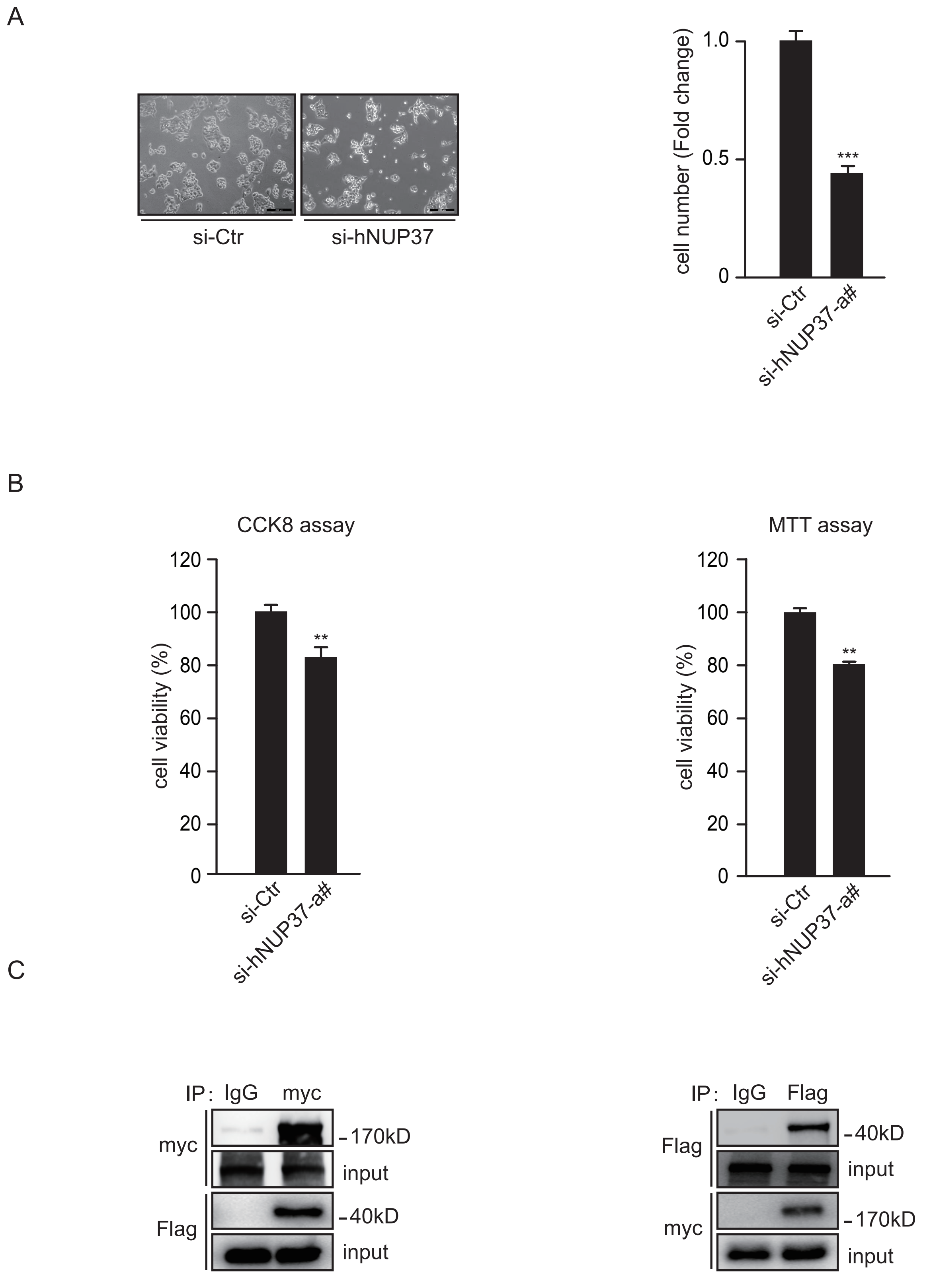
A
si-Ctr
si-hNUP37
cell number (Fold change)
1.0
0.5
0
***
si-Ctr
si-hNUP37-a#
B
CCK8 assay
cell viability (%)
120
100
80
60
40
20
0
**
si-Ctr
si-hNUP37-a#
MTT assay
cell viability (%)
120
100
80
60
40
20
0
**
si-Ctr
si-hNUP37-a#
C
IP: IgG myc
myc
170kD
input
Flag
40kD
input
IP: IgG Flag
Flag
40kD
input
myc
170kD
input

Figure 4

A

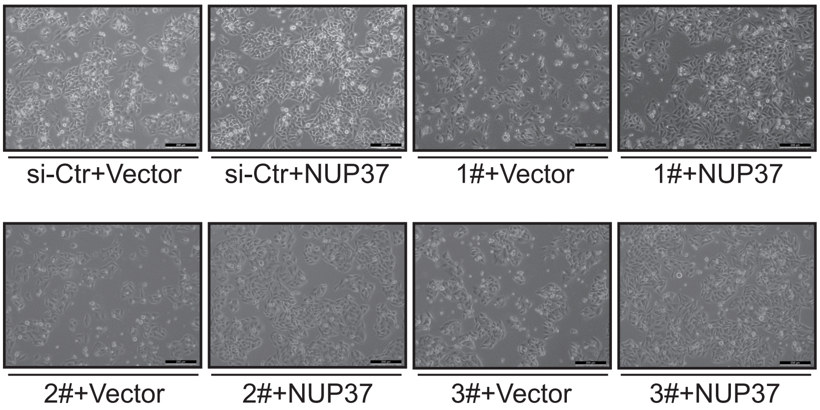
si-Ctr+Vector
si-Ctr+NUP37
1#+Vector
1#+NUP37
2#+Vector
2#+NUP37
3#+Vector
3#+NUP37

B

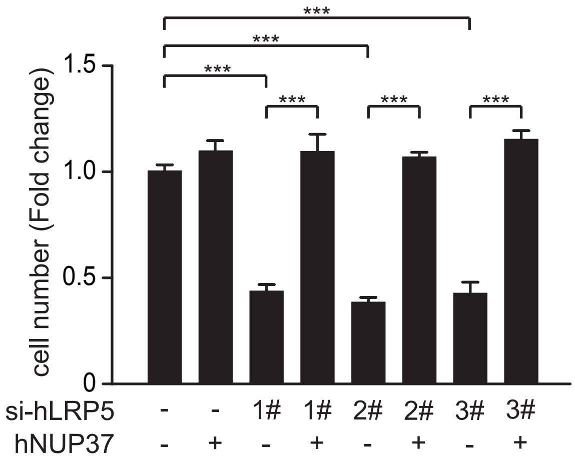
***
***
***
***
***
***
1.5
1.0
0.5
0
cell number (Fold change)
si-hLRP5 - - 1# 1# 2# 2# 3# 3#
hNUP37 - + - + - + - +

Figure 5

A

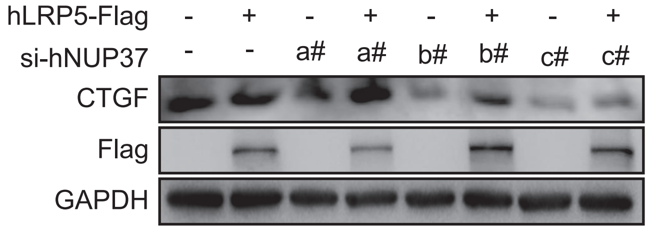
hLRP5-Flag - + - + - + - +
si-hNUP37 - - a# a# b# b# c# c#
CTGF
Flag
GAPDH

B

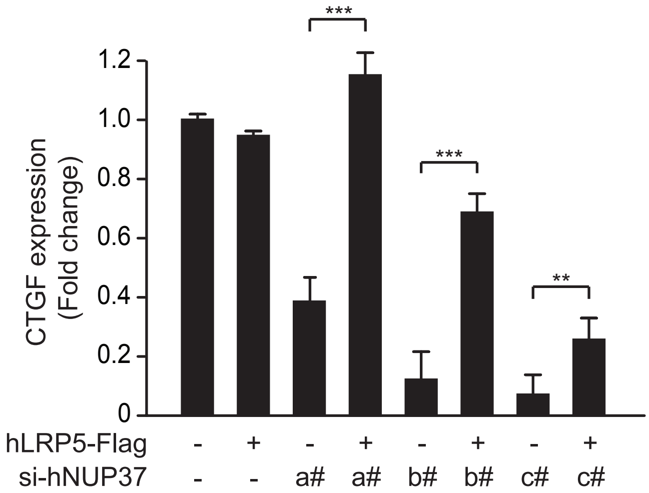
***
***
**
1.2
1.0
0.8
0.6
0.4
0.2
0
CTGF expression
(Fold change)
hLRP5-Flag - + - + - + - +
si-hNUP37 - - a# a# b# b# c# c#

Supplementary Figure 1

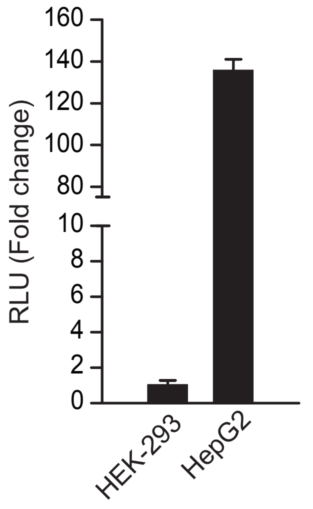

Supplementary Figure 2

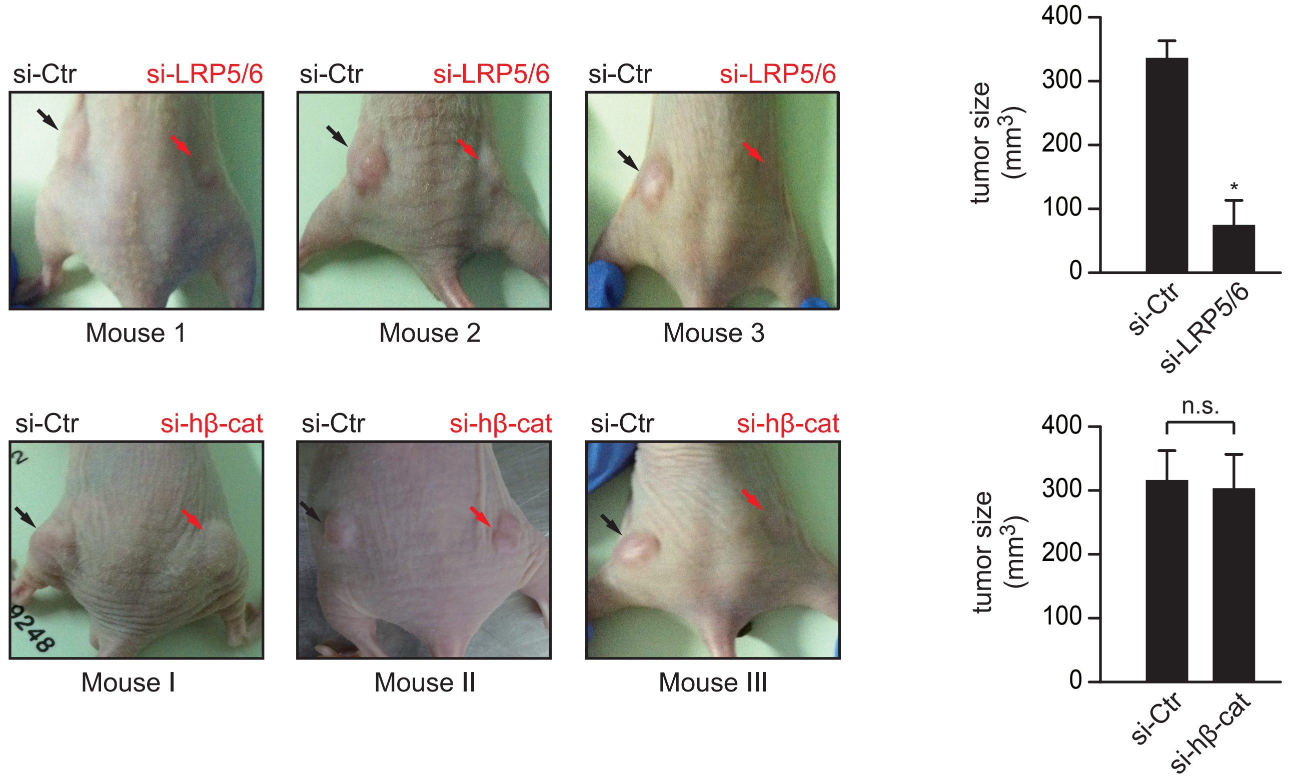

Supplementary Figure 3

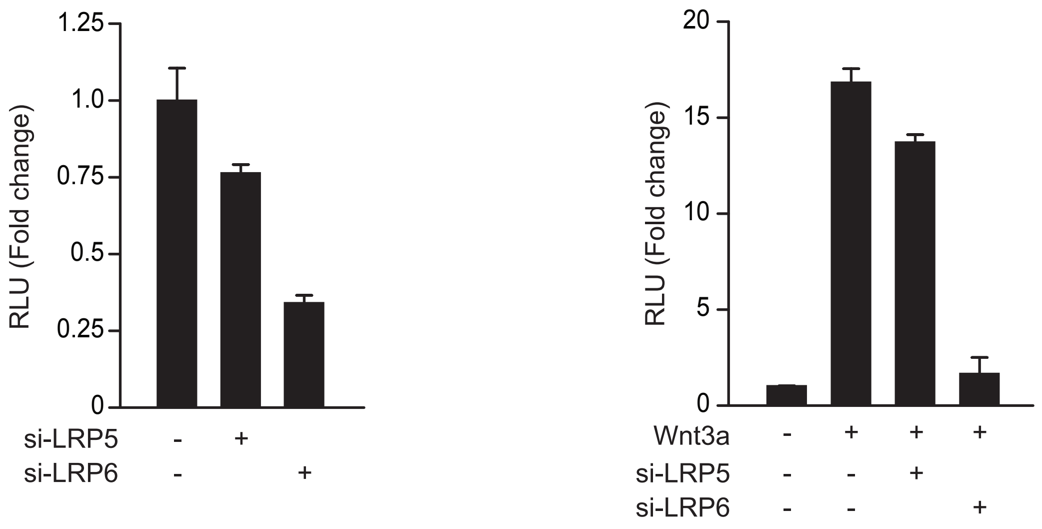

Supplementary Figure 4

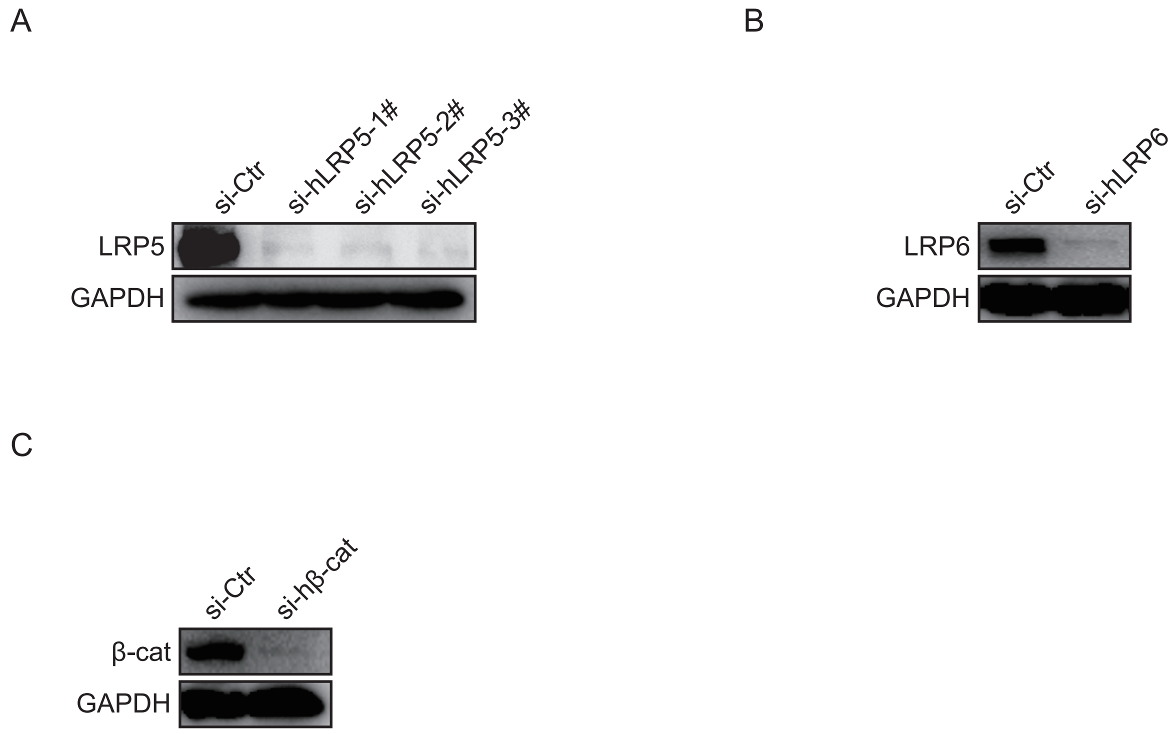

Supplementary Figure 5

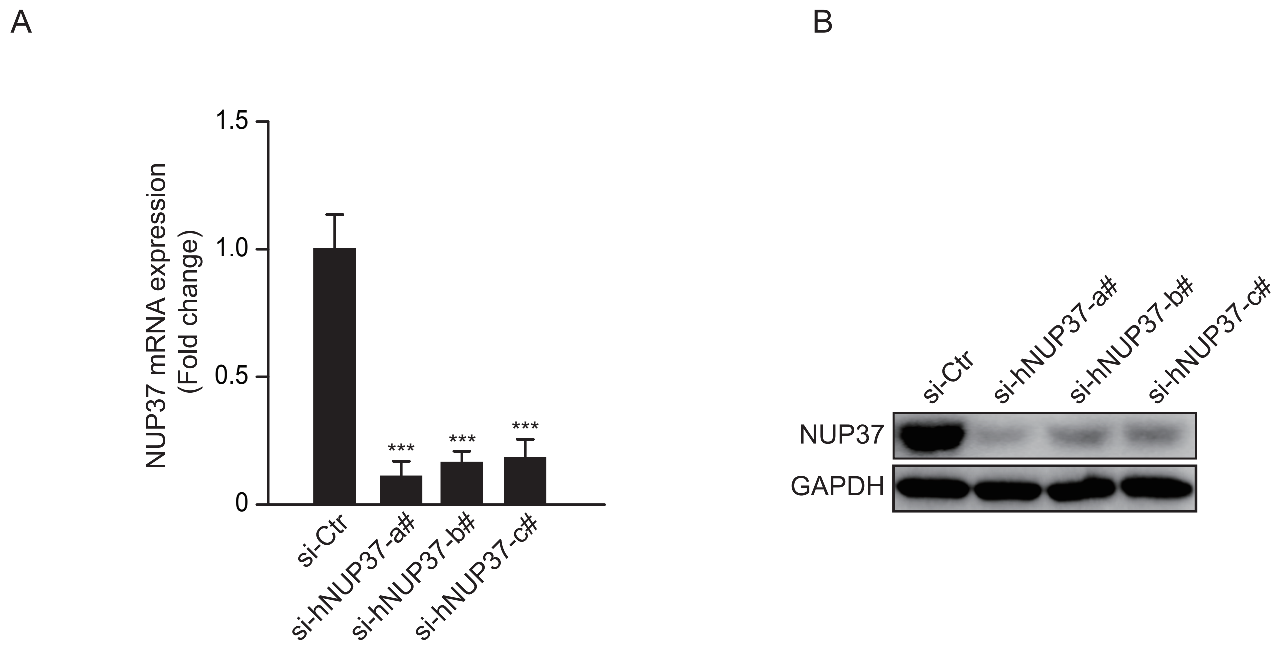

Supplementary Figure 6

A

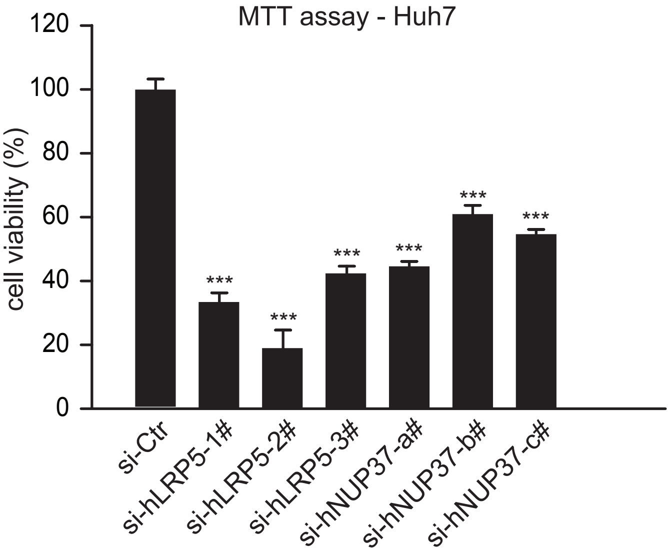

B

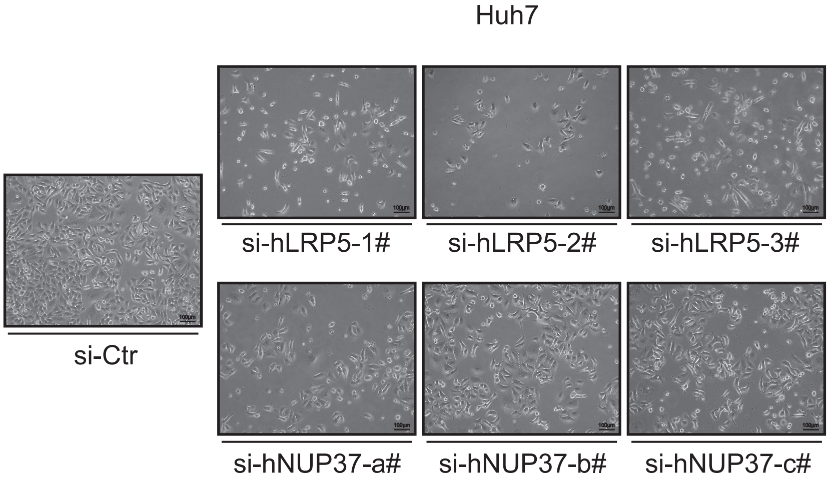

C

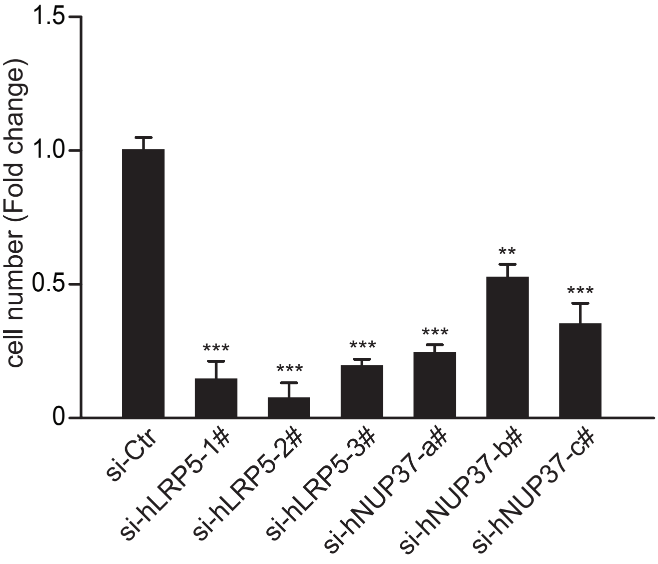

Supplementary Figure 7

A

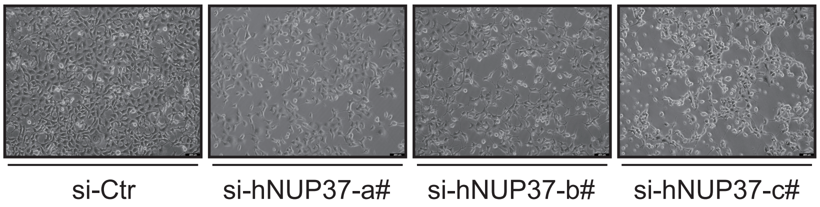

B

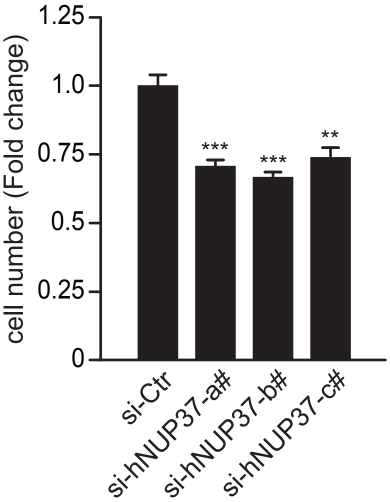

Supplementary Figure 8

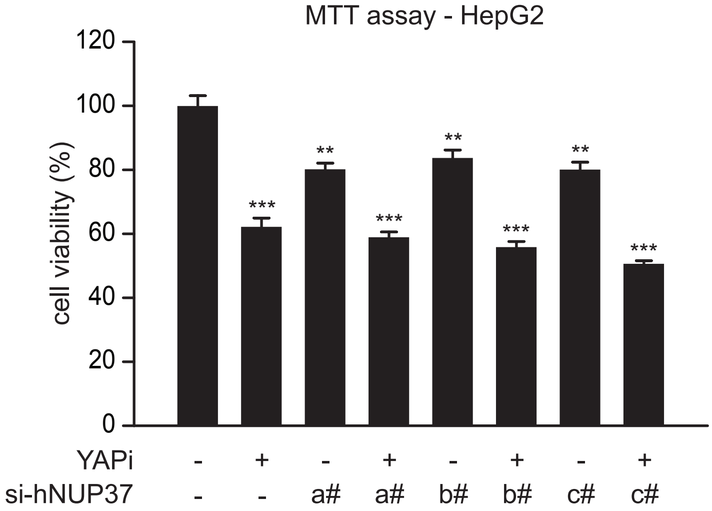

Supplementary Figure 9

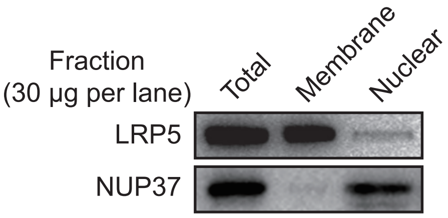

Supplement: Supplementary file 1 — Additional file 1 : Figure S1. TOPflash assay showing the basal level of Wnt/β-catenin pathway activation between HCC cell line HepG2 cells and a non cancer cell line HEK293 cells. n = 3. Figure S2. Tumor formation assay following injection of HepG2 cells in SCID/bg mice after transient transfection with control, LRP5/6, or β-catenin siRNAs. n = 4. * p < 0.05, n.s. no significance, compared to si-Ctr. Figure S3. TOPflash assay showing the individual roles of LRP5 and LRP6 in regulating Wnt/β-catenin pathway. n = 3. Figure S4. Western blots showing the knockdown efficiency of all three LRP5 siRNAs, as well as LRP6 and β-catenin siRNAs. n = 3. GAPDH, loading control. Figure S5. Knockdown efficiency of all three NUP37 siRNAs as verified using real-time PCR assay (A), as well as western blot assay (B). n = 3. GAPDH, loading control. *** p < 0.001, compared to si-Ctr. Figure S6. MTT assay (A) and Photoimages (B) of Huh7 cell proliferation following knockdown of NUP37 or LRP5. n = 3. *** p < 0.001, compared to si-Ctr. Figure S7. Photoimages and quantification of HEK-293 cell proliferation following knockdown of NUP37. n = 3. ** p < 0.01, *** p < 0.001, compared to si-Ctr. Figure S8. MTT assay showing the decrease in cell proliferation following treatment with YAP inhibitor CA3(CIL56) in NUP37 knocked-down HepG2 cells. n = 3. ** p < 0.01, *** p < 0.001, compared to si-Ctr. Figure 9. Western blots showing the subcellular localization of LRP5 and NUP37. n = 3. [file 12964_2019_495_MOESM1_ESM.pdf]
